# Sympathetic activity is correlated with satellite cell aging and myogenesis via β2-adrenoceptor

**DOI:** 10.1186/s13287-021-02571-8

**Published:** 2021-09-16

**Authors:** Shiguo Yuan, Sheng Zheng, Kai Zheng, Yanping Gao, Meixiong Chen, Yikai Li, Xiaochun Bai

**Affiliations:** 1 Guangdong Provincial Key Laboratory of Bone and Joint Degeneration Diseases, Center for Orthopaedic Surgery, The Third Affiliated Hospital, Southern Medical University, Guangzhou, 510630 China; 2 Department of Orthopaedic Surgery, Hainan Province Hospital of Traditional Chinese Medicine, Haikou, 570203 China; 3 School of Traditional Chinese Medicine, Southern Medical University, Guangzhou, 510515 China; 4 School of Basic Medical Sciences, Youjiang Medical University for Nationalities, Baise, 533000 China

**Keywords:** Skeletal satellite cells, β-Adrenoceptor, Sympathetic nerve, Clenbuterol, Skeletal muscle aging

## Abstract

**Background and objective:**

Sympathetic activity plays an important role in the proliferation and differentiation of stem cells, and it changes over time, thereby exerting differential effects on various stem cell types. Aging causes sympathetic hyperactivity in aged tissues and blunts sympathetic nerves regulation, and sympathetic abnormalities play a role in aging-related diseases. Currently, the effect of sympathetic activity on skeletal muscle stem cells, namely satellite cells (SCs), is unclear. This study aimed to investigate the effects of skeletal muscle sympathetic activity on SC aging and skeletal muscle repair.

**Materials and methods:**

To evaluate skeletal muscle and fibrotic areas, numbers of SCs and myonuclei per muscle fiber, β2-adrenoceptor (β2-ADR) expression, muscle repair, and sympathetic innervation in skeletal muscle, aged mice, young mice that underwent chemical sympathectomy (CS) were utilized. Mice with a tibialis anterior muscle injury were treated by barium chloride (BaCl_2_) and clenbuterol (CLB) in vivo. SCs or C2C12 cells were differentiated into myotubes and treated with or without CLB. Immunofluorescence, western blot, sirius red, and hematoxylin–eosin were used to evaluate SCs, myogenic repair and differentiation.

**Results:**

The number of SCs, sympathetic activity, and reparability of muscle injury in aged mice were significantly decreased, compared with those in young mice. The above characteristics of young mice that underwent CS were similar to those of aged mice. While CLB promoted the repair of muscle injury in aged and CS mice and ameliorated the reduction in the SC number and sympathetic activity, the effects of CLB on the SCs and sympathetic nerves in young mice were not significant. CLB inhibited the myogenic differentiation of C2C12 cells in vitro. We further found that NF-κB and ERK1/2 signaling pathways were activated during myogenic differentiation, and this process could be inhibited by CLB.

**Conclusion:**

Normal sympathetic activity promoted the stemness of SCs to thereby maintain a steady state. It also could maintain total and self-renewing number of SCs and maintain a quiescent state, which was correlated with skeletal SCs via β2-ADR. Normal sympathetic activity was also beneficial for the myogenic repair of injured skeletal muscle.

**Supplementary Information:**

The online version contains supplementary material available at 10.1186/s13287-021-02571-8.

## Introduction

Skeletal muscles have a good regeneration ability due to the existence of skeletal muscle stem cells, namely muscle satellite cells (SCs). Quiescent skeletal muscle stem cells are localized in the stem cell niche between the muscle membrane and the basement membrane [1]. SCs have a high myonuclear-to-cytoplasm ratio and a small number of mitochondria, which can express paired box protein 7 (PAX7) and other gene markers [2]. The nuclear-to-cytoplasmic ratio is decreased in activated SCs. Myogenic factor 5 (Myf5) expression in activated SCs is substantially increased, and MyoD expression can be detected in activated SCs [3]. MyoD and Myf5 can induce the expression of key transcription factors, such as myogenin, which can promote the differentiation of SCs/progenitor cells. The myogenin (MyoG) gene serves as a marker of early-stage differentiation [4], while myosin heavy chain and MRF4 are gene markers in the terminal stage of SC differentiation [4–6]. Myf5(−) SCs are self-renewing skeletal muscle stem cells, while Myf5(+) cells represent differentiated skeletal muscle progenitor cells [7].

SCs have a good self-renewal ability and can be activated and induced to proliferate when it's exposed to injury and exercise, which can generate new muscle cells or integrate into damaged muscle cells. However, the repair function of injured skeletal muscle in aged mice is decreased and ineffective [8]. Substantial many studies indicate that the functions of SCs change with aging, but the changes may be slight. In the SC microenvironment, changes in niche and extracellular matrix may be key factors in aging [8–10]. The niche and extracellular matrix play an important role in the maintenance, proliferation, and differentiation of stem cells.

Moreover, sympathetic activity, which plays an important role in the proliferation and differentiation of stem cells, changes over time and exerts differential effects on various stem cell types [11]. Aging causes sympathetic hyperactivity in aged mouse tissues and human colon adenoma tissues, which can blunt the sympathetic regulation of motor neuron synaptic vesicle release [12, 13]. Tyrosine hydroxylase (TH), an enzyme responsible for the rate-limiting step in catecholamine production [14], is a specific marker of sympathetic activity [15]. Neuropeptide Y (NPY) and norepinephrine (NE) are co-released by sympathetic nerves [16]. NPY is a sympathetic neurotransmitter that regulates inflammatory cells [17] and is considered to directly affect the regulation of hematopoietic stem cell fate by modulating cell quiescence [18]. NE is an important catecholamine neurotransmitter released from sympathetic nerves that can regulate the proliferation and differentiation of numerous cell types. Studies have found that sympathetic nerves of aged mice show aging-related changes, such as decreasing numbers of adrenoceptors (ADR) and sympathetic nerve density [19–21].

A short-term increase in sympathetic activity leads to an increase in skeletal muscle mass. In contrast, chronic sympathetic hyperfunction is harmful, leading to heart failure-induced skeletal muscle myopathy [22]. In previous studies, we found that in myofascial pain syndrome, the most common chronic skeletal muscle disease, the formation of myofascial trigger points led to local sympathetic hyperactivation, thus hindering the repair process [23]. However, pretreatment via chemical sympathectomy (CS), which inhibits the growth of muscle fibers and affects the local inflammatory state, did not promote skeletal muscle repair in myofascial pain syndrome [24]. After CS, the increased sympathetic activity at myofascial trigger points was decreased, with skeletal muscle repaired and the number of SCs restored [25]. Sympathetic activity is decreased in aged mice, and the niche that maintains hematopoietic stem cells is affected, leading to aging-related effects [19]. We hypothesize that sympathetic nerves may be a component of the SC niche and that normal activity helps maintain the stemness of SCs and promotes repair after injury.

Therefore, using a mouse model of CS-induced denervation to simulate the low sympathetic innervation state of aged mice, we detected sympathetic innervation, the number of SCs, and the changes in repair levels after muscle injury in aged mice. Skeletal muscle was injured by barium chloride (BaCl_2_) and treated with a sympathetic β-ADR agonist, clenbuterol (CLB), to observe the changes in repair levels after skeletal muscle injury. CLB triggers skeletal muscle hypertrophy by promoting protein synthesis to thereby reduce rodent muscle atrophy due to neuromuscular damage caused by factors such as denervation [26]. This study aimed to explore the effects of sympathetic nerves on SCs and skeletal muscle injury repair in young and aged mice.

## Materials and methods

### Mice modeling and treatment

Ten-month- or 6-week-old C57BL/6 J mice (50% male mice and 50% female mice) were purchased from the Animal Experimental Center of Guangzhou University of Chinese Medicine. Ten-month-old mice were routinely reared to 18 months of age. All animal experiments were approved by the Animal Ethics Committee of Southern Medical University. The mice were randomly divided into six groups: control group, injury group, CS group, CLB group, CS + injury group, and CS + injury + CLB group (*n* = 8/group). The mice in the injury group were injected intramuscularly with 50 μl of sterile 1.2% BaCl_2_ (Macklin, 10361-37-2, China) using an insulin syringe (Kindly, U-40-B, 30G 0.3*8 mm needle, China) into the proximal end of the tibialis anterior (TA) muscle as previously described [27, 28]. To explore the role of sympathetic nerves, the mice in the CS group were subjected to CS via an intraperitoneal injection of 0.1 mg/g·day 6-hydroxydopamine hydrochloride (6-OHDA, Yuanye Bio-Technology, S30042-1g, China) for 5 consecutive days, followed by one injection every 6 days [29–31]. The mice in the CLB group received an intraperitoneal injection of 1 mg/kg CLB (Solarbio, YZ-100072, China) daily until killing to activate β2-ADR [32, 33]. The mice in the CS + injury group received 6-OHDA for 5 days and then injected BaCl_2_ once in the TA muscle to induce muscle injury followed by one dose of 6-OHDA every 6 days. The mice in the CS + injury + CLB group received 6-OHDA for 5 days and then injected BaCl_2_ once followed by 6-OHDA every 6 days and CLB daily. The mice were killed on days 3, 10, 21, and 42 after injury to detect changes in sympathetic nerves, SCs, and myogenic repair levels.

### SCs

SCs, the stem cells of skeletal muscle, were extracted from the TA muscles of the mice. As previously described, the mouse's TA muscles were dissected under sterile conditions and minced after the blood vessels, connective tissues, and adipogenic tissues were removed [34, 35], and then stirred in digestion buffer (0.7% collagenase II in 10% fetal bovine serum [FBS, Gibco, 10099-141, USA) in Dulbecco’s modified Eagle’s medium (DMEM)] at 37 °C for 30 min, followed by centrifugation and filtration [36]. A single-cell suspension was prepared and purified using magnetic-activated cell sorting with a Satellite Cell Isolation Kit (Miltenyi Biotec™, 130-104-268, Germany) according to the manufacturer’s instructions [36]. Extracted SCs were cultured with myoblast growth medium [DMEM/F12 (Gibco, DMEM-F12, USA) supplemented with 10% FBS and 5 ng/mL fibroblast growth factor 2 (GLPBIO, GP20218, USA)] [37]. PAX7, MyoD, MyoG, and Myf5 were used to analyze the state of SCs. Pax7(+) nuclei were considered to be SCs, whereas MyoD(+) Pax7(+) nuclei were considered to be activated for myogenic differentiation, and Myf5(−) Pax7(+) nuclei were considered to be self-renewing SCs.

### C2C12 cells

Myoblast C2C12 cells were purchased from the Kunming Cell Bank of Chinese Academy of Sciences (KCB2012115YJ, China) and cultured in high-glucose DMEM supplemented with 10% FBS [38]. C2C12 cell differentiation into myotubes was conducted in high-glucose DMEM supplemented with 2% horse serum upon reaching 80% confluence. Cells in the differentiation + CLB group were cultured in high-glucose DMEM supplemented with 2% horse serum and CLB (10 μM and 100 μM) to 80% confluence to observe the changes in the SC and myogenic ability markers.

### Histology

Mouse TA muscles were isolated, fixed, dehydrated, embedded, and cut into sections with a standard method. Slides were stained with Sirius red (SR, Tiandz, 121104-100, China) and hematoxylin–eosin (G1120, Solarbio, China) according to the manufacturer’s instructions. The areas of muscle cells and fibrosis were quantified using ImageJ software. Additionally, the number of myonuclei per muscle fiber was determined and analyzed.

### Immunofluorescence

Immunofluorescence staining was conducted to detect markers of SCs and ADR. Slides were deparaffinized in xylene, rehydrated, and blocked with bovine serum albumin for 2 h at room temperature. Cells on confocal dishes were fixed with 4% paraformaldehyde, and the confocal dishes or slides were incubated with 0.1% Triton X-100 and primary antibodies at a dilution of 1:50–1:200 for approximately 16 h at 4 °C. The primary antibodies were as follows: PAX7 (sc-81648, Santa Cruz, USA; DF7915, Affinity, USA), MyoD (AF7733, Affinity, USA), Myf5 (39801, Active Motif, USA), TH (511027, Zen Bio, China), NPY (ab112473, Abcam, UK), β2-ADR (bs-0947R, Bioss, China), and MYH (sc-376157, Santa Cruz, USA). The slides were incubated with secondary antibodies (KGAB010, KGAB011, KGAB013, KGAB014, Keygen, China) at room temperature for 2 h at a 1:400 dilution, sealed with DAPI and observed by a confocal scanning microscope (Olympus, FV1200, Japan) or a fluorescence microscope (Konigsallee 9–21, Carl Zeiss Microscopy GmbH, Germany). The fluorescence intensity was quantified using FV10-ASW 4.2 Viewer or ImageJ software.

### Western blot analysis

Total proteins were extracted from SCs at 24 h after isolation or from C2C12 cells at 48 h after treatment using SDS-PAGE, transferred onto nitrocellulose membranes and incubated with primary antibodies for approximately 12 h at 4 °C. The primary antibodies were as follows: adrenergic receptor (bs-0498P, bs-1062P, bs-1063R, Bioss, 1:500, China), nuclear factor-κB (NF-κB) signaling pathway proteins (9936T, CST, 1:1000, USA), ERK1/2 (4695T, CST, 1:1000, USA), JNK (24164-1-AP, Proteintech, 1:1000, USA) (14064-1-AP, Proteintech, 1:1000, USA), and the marker of SC cells as described above (all diluted at 1:1000). Proteins were visualized by autoradiography and analyzed using GENE Sys V1.5.2.0.

### Enzyme-linked immunosorbent assay (ELISA)

Muscle NE was detected by ELISA. The tissue homogenate was centrifuged at 10,000 r/min for 30 min, and the supernatant was centrifuged at 12,000 r/min for 60 min. All samples were assessed in accordance with the instructions of the ELISA kit (JM-02907M1, Yibai Technology Co., Ltd., China). Curve Expert 2.20 software was used to fit the standard curve, and the values of each index were calculated for statistical analysis. All samples were assessed in duplicate.

### Statistical analysis

All data were presented as the mean ± standard error. SPSS (version 17.0, Inc., Chicago, USA) was used for statistical analyses, and a *P* value < 0.05 was considered significant. Independent samples *t* tests were used for pairwise comparisons between two groups. One-way ANOVA with Tukey’s test was used to assess the significant differences among more than two data groups.

## Results

### Skeletal muscle fibrosis was increased, while the number of SCs and sympathetic activity were decreased in aged mice

**Fig. 1:**
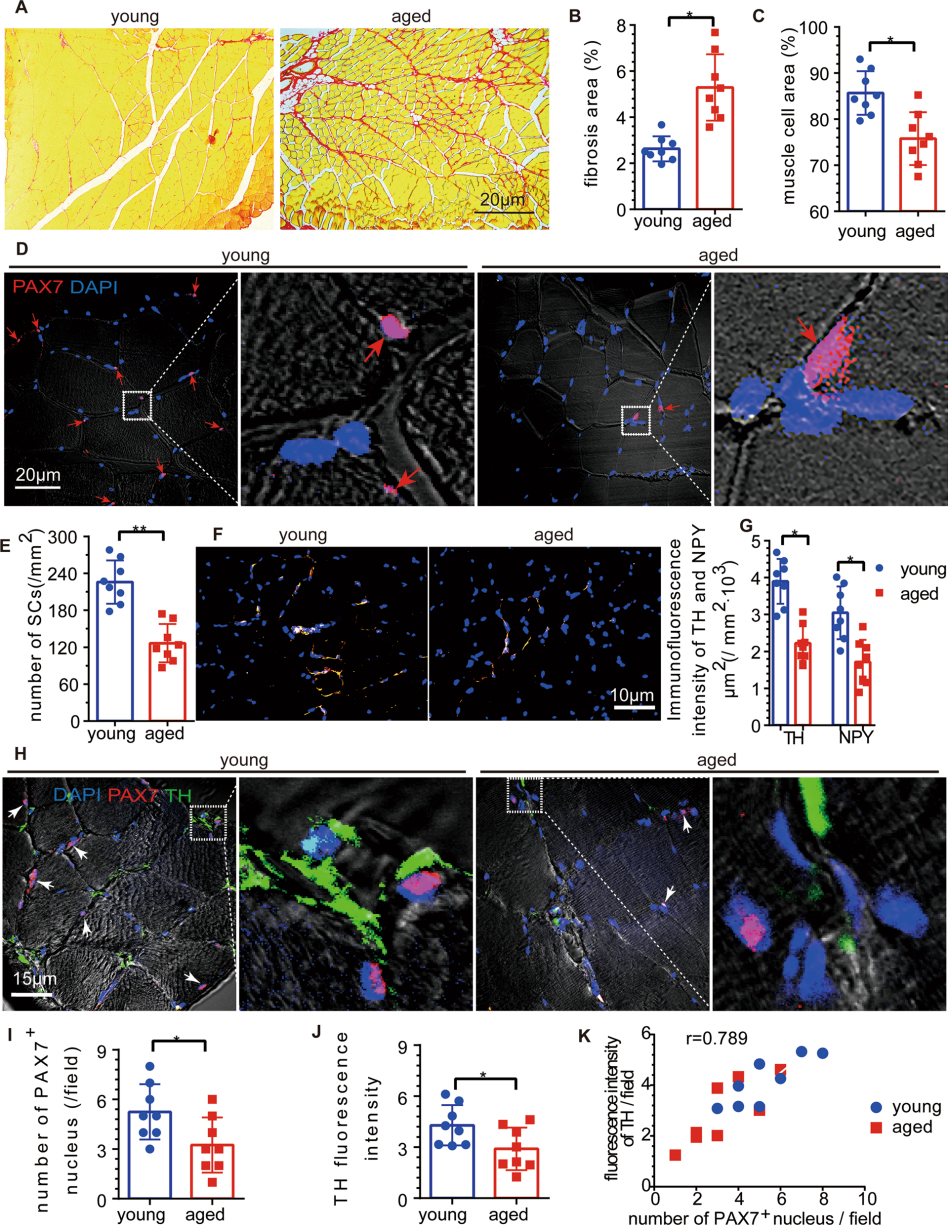
The skeletal muscle fibrotic area was increased in aged mice, while the SC numbers and sympathetic nerve activity were decreased. **A**–**C** SR staining showed that the proportion of the fibrotic area was increased and that the proportion of the myocyte area was decreased in the aged mice (*n* = 8) (*t* = 4.89 and 3.77, *P* < 0.001 and 0.021). **D**, **E** Immunofluorescence staining showed that the number of PAX7^+^ nuclei (SCs, red arrow) was decreased significantly in aged mice (*n* = 8) (*t* = 5.97, *P* < 0.001). **F**, **G** Immunofluorescence staining showed that the levels of TH and NPY in the sympathetic nerves of the aged mice were significantly lower than those of the young mice (*n* = 8) (*t* = 1.54 and 2.07, *P* = 0.017 and 0.008). **H**–**K** Both the intensity of TH and the number of PAX7^+^ nuclei (SCs, white arrow) were decreased significantly in aged mice compared with young mice (*n* = 8 per group) (*t* = 2.28 and 2.40, *P* = 0.039 and 0.031), and the intensity of TH correlated with the number of PAX7^+^ nuclei (*r* = 0.789, *P* < 0.001). Independent samples *t* tests, *P < 0.05; **P < 0.001

First, we found that muscle cells of the young mice were more compact, with larger area of the muscle cells, and lower proportion of fibrous tissue, compared with the aged mice (Fig. 1A–C). Then, we investigated whether the SCs exhibited the corresponding changes. Compared with that in young mice, the absolute number of SCs (PAX7(+) nuclei) in the skeletal muscles of aged mice was significantly decreased (Fig. 1D, E), which was consistent with previous studies [39]. However, the number of myonuclei per muscle fiber was not significantly different among the NC (young mice), CS, CLB, and CS + CLB groups (Additional file 1: Fig. S1A, B).

### The sympathetic innervation of aged mice was weakened

**Fig. 2:**
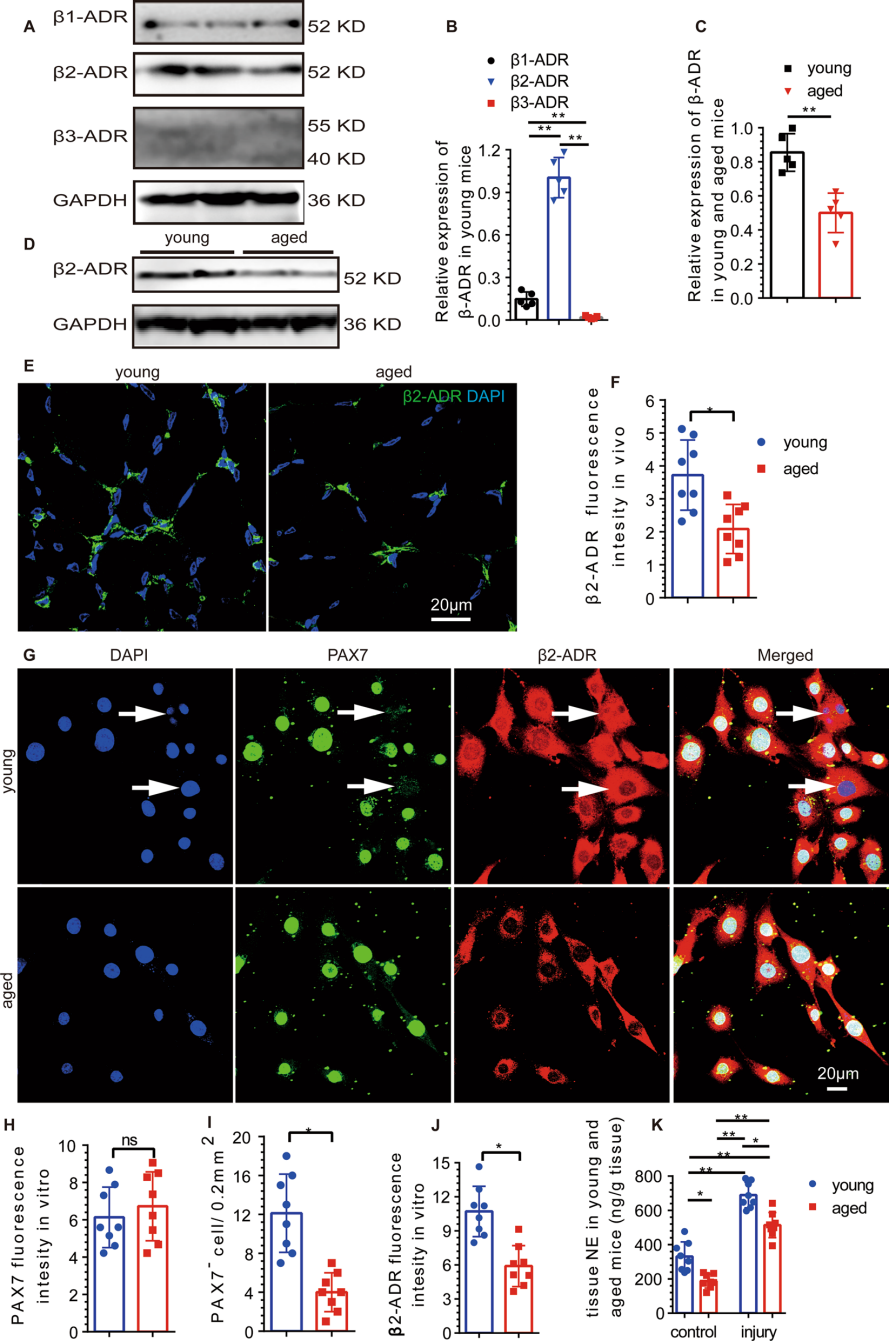
The distributions of β-ADR and β2-ADR were decreased in aged SCs. **A**, **B** WB analysis showed low expression of β1-ADR, high expression of β2-ADR, and almost no expression of β3-ADR (*n* = 5) (*F* = 18.95, *P* < 0.001). **C**, **D** The β2-ADR expression in the SCs of aged mice was significantly lower than that in the SCs of young mice (*n* = 5) (*t* = 6.321, *P* < 0.001). **E**, **F** Immunofluorescence staining showed that the expression of β2-ADR in the TA muscles of aged mice was lower than that in the TA muscles of young mice (*n* = 8) (*t* = 1.31, *P* = 0.014). **G**–**J** Immunofluorescence staining showed that the expression of PAX7 was not significantly decreased in the SCs of aged mice in vitro (*n* = 8) (*t* = 0.18, *P* = 0.25) but that the number of PAX7^−^ SCs (white arrow) in the proliferative phase was decreased significantly (*n* = 8) (*t* = 2.08, *P* = 0.007). The expression of β2-ADR was lower in aged mice than that in young mice (*n* = 8) (*t* = 1.67, *P* = 0.011). **K** The tissue concentration of NE was higher in young mice than in aged mice in both the noninjury and injury states (*t* = 3.07 and 2.97, *P* = 0.009 and 0.012). Independent samples *t* tests and one-way ANOVA with Tukey’s test, ns, not significant, *P < 0.05; **P < 0.001

We further examined sympathetic innervation in TA muscle. We found that sympathetic activity was decreased significantly in aged mice, which was consistent with the decreasing trends in the muscle cell area and SC number (Fig. 1F, G). Both the fiber density of sympathetic nerves and the SC (Pax7(+) nuclei) density were decreased in aged mice (Fig. 1H–J), and the fiber density of sympathetic nerves was correlated with the density of SCs in young and aged mice (Fig. 1H, K). β2-ADR was shown to be expressed in skeletal muscle [40, 41], and β1 and β2-ADR were expressed in C2C12 cells (a mouse skeletal muscle stem cell line) [38]. Next, we determined the expression of ADR in SCs. SCs were extracted and purified, and the expression of β-ADR was measured by WB analysis. Low expression of β1-ADR, highest expression of β2-ADR, and barely expressed β3-ADR were found in SCs from young mice (Fig. 2A, B). Moreover, the β2-ADR expression in SCs from aged mice was decreased compared with that in SCs from young mice (Fig. 2C, D). The expression of β2-ADR in young mice was higher than that in aged mice in vivo (Fig. 2E, F). SCs were extracted, purified, and cultured in vitro, and the expression of β2-ADR in SCs from aged mice was lower than that in SCs from young mice. The in vitro fluorescence intensity of PAX7 did not significantly differ between the aged and young mice (Fig. 2G, H). Proliferating SCs, which were undergoing mitosis, were more abundant in young mice than that in aged mice (Fig. 2G, I). The expression of β2-ADR was decreased in the SCs of aged mice (Fig. 2G, J).

### NE was less abundant in TA muscles, and myogenic repair was impaired in aged mice

**Fig. 3:**
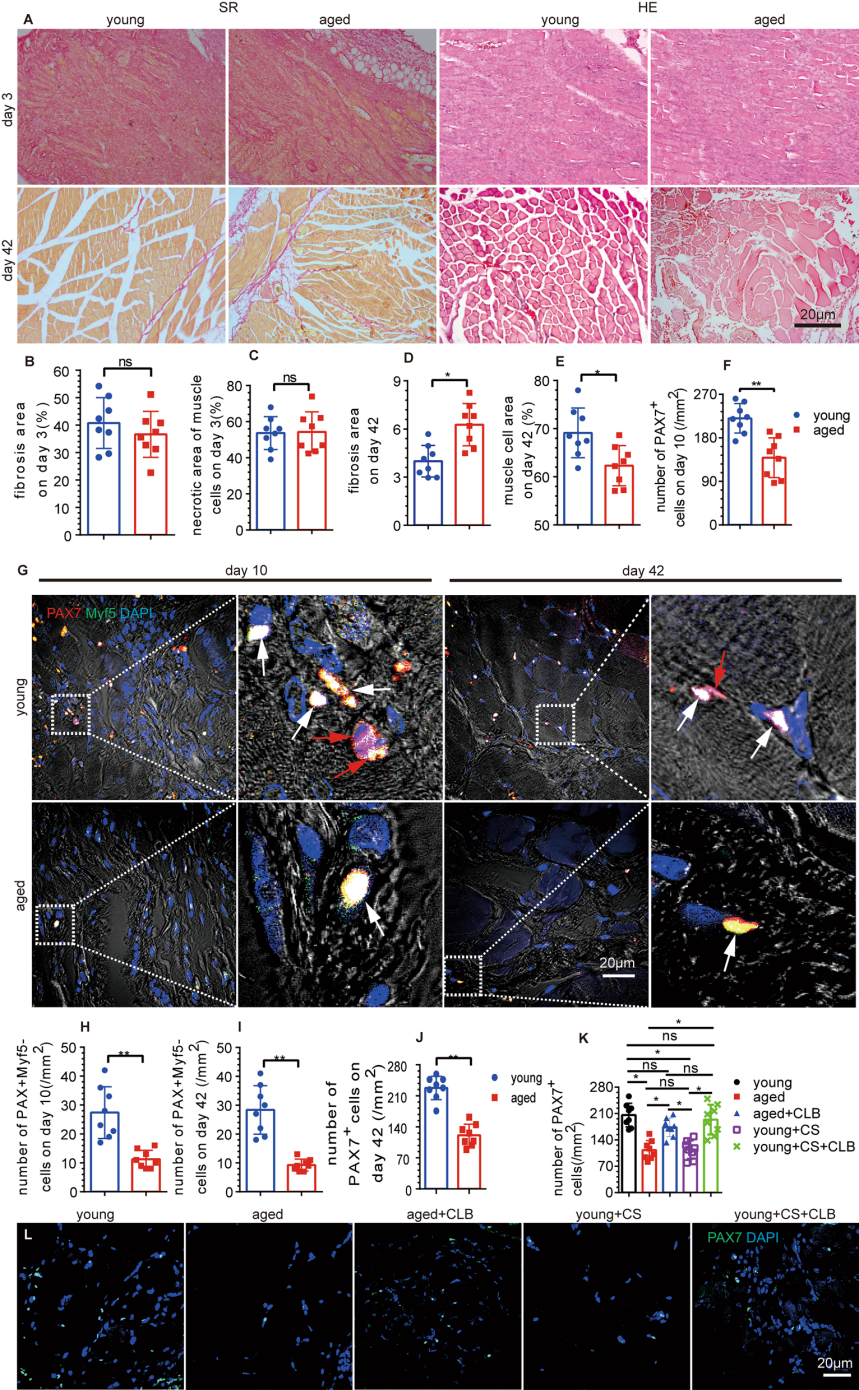
Skeletal muscle repair and the number of SCs were reduced in aged mice. **A**–**C** The proportions of fibrotic and necrotic muscle cells were not significantly different between the aged and young mice on the 3rd day after TA injury (*n* = 8) (*t* = 0.931 and 0.116, *P* = 0.368 and 0.910). **D**, **E** On the 42nd day after injury, the proportion of fibrosis in the aged mice was higher than that in the young mice, and the muscle cell area in the aged mice was smaller than that in the young mice (*n* = 8) (*t* = 3.911 and 2.897, *P* = 0.002 and 0.012). **F**–**J** The numbers of PAX7^+^ SCs (white and red arrow) and self-renewing PAX7^+^Myf5^−^ SCs (red arrow) were decreased substantially in aged mice compared with young mice on days 10 (*n* = 8) (*t* = 8.341 and 6.24, both *P* < 0.001) and 42 (*n* = 8) (*t* = 9.815 and 4.825, both *P* < 0.001) after injury. **K**, **L** The number of PAX7^+^ (SCs) in aged mice and CS-treated mice was reduced, and CLB partially reversed this decrease (*F* = 14.912, *P* < 0.001). Independent samples *t* tests and one-way ANOVA with Tukey’s test, ns, not significant, *P < 0.05; **P < 0.001

NE from TA muscles was further detected, and its expression was decreased in aged mice compared with young mice. Moreover, the NE expression was increased to a lesser extent in the aged mice than in the young mice at 3 days after injury induced by BaCl_2_ (Fig. 2K). After local injection of BaCl_2_ into the TA muscle, extensive muscle cell necrosis, numerous fibrosis-positive areas, and inflammatory cell infiltration were observed in aged mice. There was no significant difference between the young mice and aged mice at 3 days after injury (Fig. 3A–C). The number of newly formed myotubes in aged mice was significantly lower than that in young mice. The positive area of fibrosis staining was much larger in aged mice than in young mice at 10 days after the injection (Additional file 1: Fig. S1C, E). On day 21, young mice showed good recovery; however, the aged mice had significantly fewer muscle cells than the young mice with increased fibers (Additional file 1: Fig. S1D, G). On day 42, the myocytes of the young mice exhibited an orderly arrangement and a regular polygonal shape, while the aged mice showed disordered, rounded, or round-like myocytes. Moreover, the fibrotic tissue area was greater in the aged mice than in the young mice, but the myocyte area was smaller (Fig. 3A, D, E).

### The number of SCs in aged mice was further decreased after injury and rescued by CLB in SCs

The number of SCs was decreased in the aged mice on day 10 after injury (Fig. 3F, G). Additionally, the number of self-renewing SCs (detected by Myf5(−) PAX7(+)) in aged mice was significantly lower than that in young mice at 10 days after BaCl_2_-induced injury (Fig. 3G, H). After 42 days, the numbers of SCs and self-renewing SCs were further reduced (Fig. 3G, I, J). After CLB supplementation, the number of SCs was significantly increased in TA muscles of aged mice (Fig. 3K, L).

### Sympathetic hypoinnervation was observed in mice that underwent CS and was rescued by CLB

**Fig. 4:**
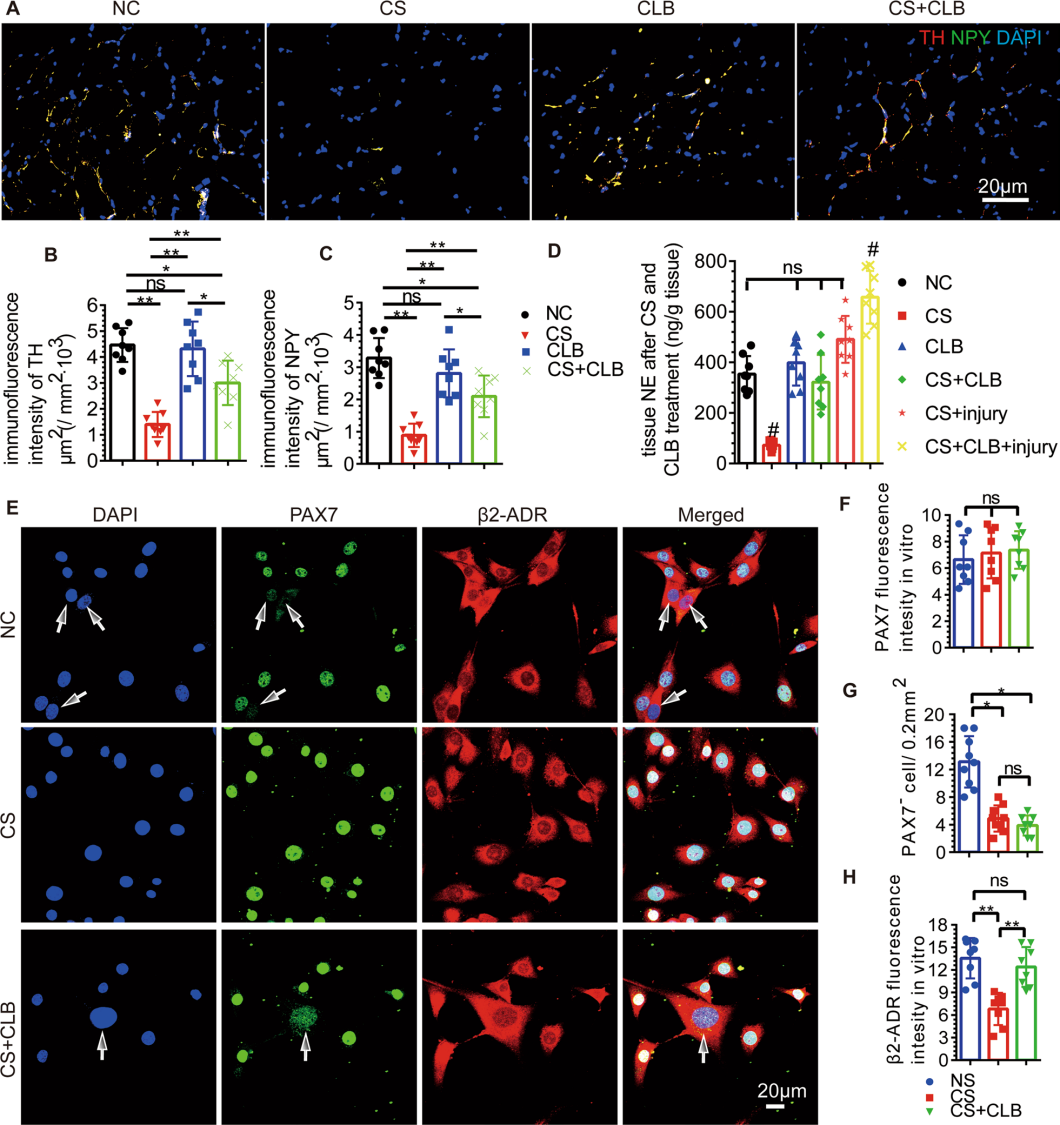
CLB partly rescued sympathetic hypoinnervation and increased the expression of β2-ADR in SCs after CS. **A**–**C** The decreased expression of TH and NPY, which indicates sympathetic hypoinnervation, was partly reversed by CLB, but their expression levels were not changed in normal mice treated with only CLB (*F* = 39.989 and 23.328, both *P* < 0.001). **D** The tissue expression of NE was decreased after CS, and CLB increased the NE expression in the CS group; however, the increase was not as extensive as that in the CS + CLB + injury group (*F* = 44.23, *P* < 0.001). **E**–**H** CS reduced the expression of β2-ADR and the proliferation of SCs (white arrow), which was partially rescued partly by CLB (*F* = 16.642, 12.447, both *P* < 0.001). One-way ANOVA and Tukey’s test, ns, not significant, ^#^all *P* < 0.05 compared with all other groups, *P < 0.05, **P < 0.001

The fluorescence intensities of TH and NPY were decreased significantly after CS (Fig. 4A–C) and then increased after CLB treatment. However, the fluorescence intensities of TH and NPY in the young mice did not increase significantly after CLB treatment, indicating that the sympathetic nerves were significantly damaged after CS and CLB protected against sympathetic nerve damage (Fig. 4A–C). Furthermore, the level of NE was significantly decreased in the mice after CS, and this effect was rescued by CLB (CS + CLB group) (Fig. 4D). There was no significant increase in NE in the young mice after the CLB injection (CLB group) (Fig. 4D). The change in NE was consistent with the sympathetic activity.

### The expression of β2-ADR in SCs was reduced, and SCs differentiation was induced

**Fig. 5:**
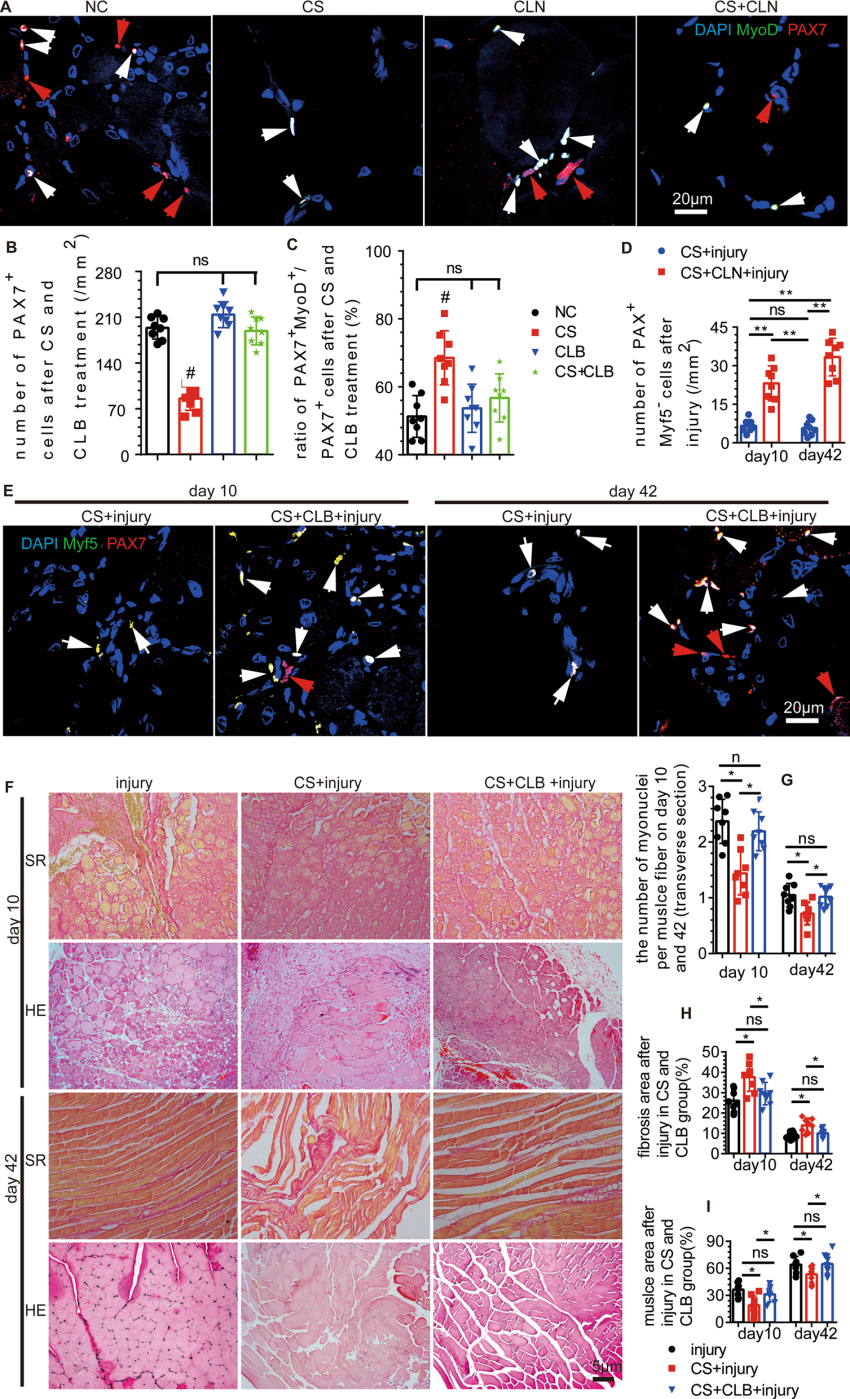
CLB promoted skeletal muscle repair by affecting MyoD and Myf5. **A**–**C** CS decreased the number of PAX7^+^ cells (SCs), but the ratio of MyoD^+^/MyoD^+^PAX7^+^ cells was increased significantly. That is, CS led to the activation of SCs, which hindered their ability to maintain their stemness. CS + CLB treatment prevented this trend. In addition, the PAX7^+^ cell level and the ratio of MyoD^+^/MyoD^+^ PAX7^+^ cells were not significantly changed by CLB alone in normal mice (*F* = 15.363 and 5.911, *P* < 0.001 and = 0.03). **D**, **E** On the 10th and 42nd days after injury, the number of Myf5^−^ PAX7^+^ cells (SCs) in the CS + CLB + injury group was significantly increased compared with that in the CS + injury group (*F* = 51.219, *P* < 0.001). **F**–**I** On the 10th and 42nd days after injury, the CS + injury group had the lowest number of myonuclei per muscle fiber (transverse section), and this effect was rescued by CLB. The CS + injury group had the largest fibrotic area (*F* = 11.644, 16.573, both *P* < 0.001) and the smallest muscle area (*F* = 11.644, 16.573, both *P* < 0.001), and this effect was rescued by CLB on both the 10th and 42nd days after injury. One-way ANOVA and Tukey’s test, ns, not significant, ^#^ all *P* < 0.05 compared with all other groups *P < 0.05; **P < 0.001

We found that the expression of β2-ADR in CS group was decreased after extraction and cultured for 12 h (Fig. 4E, H), and the number of SCs was significantly decreased in the proliferative phase. However, the expression of β2-ADR was increased in the CS + CLB group, compared with that in the CS group, and the expression of PAX7 was not significantly different among the NC, CS, and CS + CLB groups after extraction and cultured for 12 h in vitro (Fig. 4E–H). SC numbers were decreased, and the ratio of MyoD(+) PAX7(+)/PAX7(+) SCs was increased after CS (Fig. 5A–C). While the number of PAX7(+) SCs was increased in the CS + CLB group, the ratio of MyoD(+) PAX7(+)/PAX7(+) SCs was decreased significantly, compared with that in the CS group (Fig. 5A–C). The number of PAX7(+) SCs and the ratio of MyoD(+) PAX7(+)/PAX7(+) SCs did not change significantly after CLB injection in young mice (CLB group), compared with those in NC group (Fig. 5A–C).

### CLB supplementation helped maintain SC self-renewal and promoted myogenic repair in injured mice that underwent CS

Ten days after BaCl_2_ injury, the number of self-renewing SCs (Myf5(−) PAX7(+)) in the CS + injury group was decreased significantly. However, CLB supplementation in the CS + CLB + injury group helped maintain the number of self-renewing SCs on days 10 and 42 after injury (Fig. 5D, E). Extensive muscle cell necrosis, numerous fibrosis-positive areas, and extensive inflammatory cell infiltration were found on day 3 after muscle injury (Additional file 2: Fig. S2A–C). The muscle area and fibrosis area were not significantly different among the injury, CS + injury, and CS + CLB + injury groups on day 3 after muscle injury (Additional file 2: Fig. S2A–C). In the injury group, many nuclei were found in the center of the cells after 10 days, which indicated cell regeneration. Correspondingly, the number of newly formed myocytes in the CS + injury group was significantly lower than that in the injury group, and CLB significantly rescued this trend in the CS + injury + CLB group at 10 days after injury (Fig. 5F–H). Furthermore, the positive fibrotic area in the CS + injury group was the largest among the three groups at 10 days after injury (Fig. 5F, G, H). On day 21 after BaCl_2_ injection, the percentage of muscle cells in the injury group was the highest among these three groups (Additional file 2: Fig. S2A, C). Among the three groups, while the repair was increased in every group, the fibrotic area was still the largest in the CS + injury group, and the muscle cell area was still the smallest in the CS + injury group on day 42 after BaCl_2_ injury. The number of myonuclei per muscle fiber was decreased in the CS + injury group, which was rescued by CLB on days 10, 21, and 42 (Fig. 5I, Additional file 2: Fig. S2A, B). The fibrotic and muscle cell areas were not significantly different between the injury group and CS + CLB + injury group on day 42 after injury (Fig. 5F–H).

### C2C12 cell activation and myogenic differentiation were inhibited by CLB in vitro

**Fig. 6:**
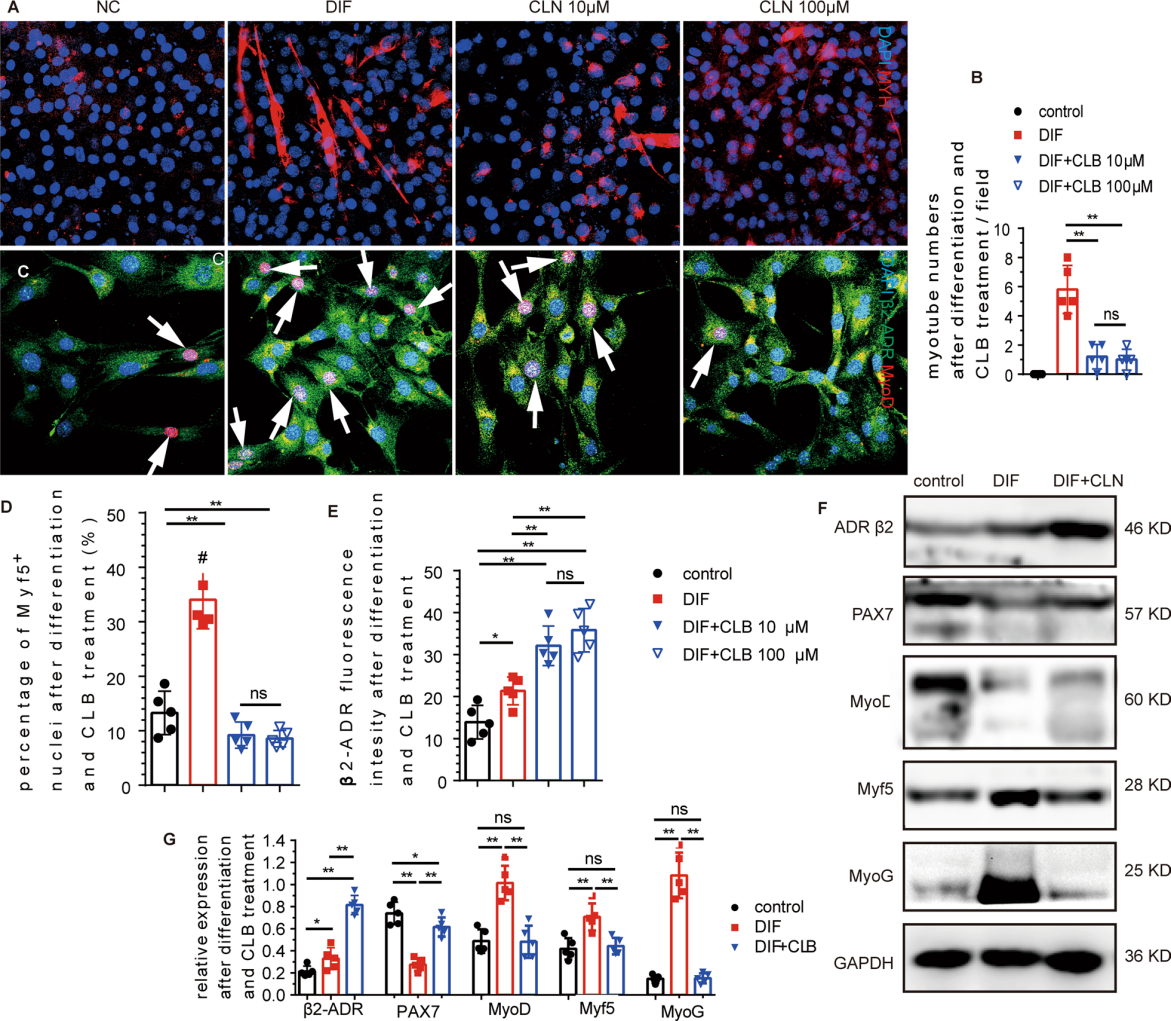
CLB-inhibited myogenic differentiation. **A**, **B** After 5 days of differentiation, numerous fused myotubes were observed, while CLB (10 μm, 100 μm) significantly inhibited myotube formation (*F* = 28.9, *P* < 0.001). **C**–**E** After 48 h of myogenic differentiation, the levels of MyoD^+^ nuclei and β2-ADR were significantly increased, while CLB significantly decreased the expression of MyoD and further increased the expression of β2-ADR (*F* = 9.642, 8.157, both *P* < 0.001). **F**, **G** After 48 h of differentiation, β2-ADR expression was increased, and CLB further promoted this increase (*F* = 115.712, *P* < 0.001). PAX7 expression was decreased after differentiation, and CLB reversed this trend (*F* = 18.155, *P* < 0.001). MyoD, Myf5, and MyoG expressions were increased significantly after differentiation, and CLB significantly mitigated these increments (*F* = 16.324, 8.647, and 154.647, all *P* < 0.001). All differences were determined using one-way ANOVA and Tukey’s test. ns, not significant, *P < 0.01; **P < 0.001

Immunofluorescence staining showed that myotubes formed and fused at 5 days after differentiation in vitro. Both 10 μM and 100 μM CLB significantly inhibited myogenic differentiation (Fig. 6A, B). After 48 h of myogenic differentiation in vitro, the expression of MyoD and β2-ADR was increased significantly, indicating that myogenic differentiation was activated. Interestingly, although 10 μM and 100 μM CLB further promoted the expression of β2-ADR, CLB inhibited the expression of MyoD and thus inhibited the activation of C2C12 cells (Fig. 6C–E). Moreover, we measured the expression of relevant proteins and found that PAX7 was significantly decreased at 48 h after myogenic differentiation and increased after 100 μM CLB treatment (Fig. 6F, G). β2-ADR expression was increased after 48 h of differentiation and further increased after 100 μM CLB treatment (Fig. 6F, G). The MyoD, Myf5, and MyoG levels were significantly increased after differentiation, which were rescued by 100 μM CLB (Fig. 6F, G).

### The NF-κB and ERK1/2 signaling pathways were inhibited by CLB

**Fig. 7:**
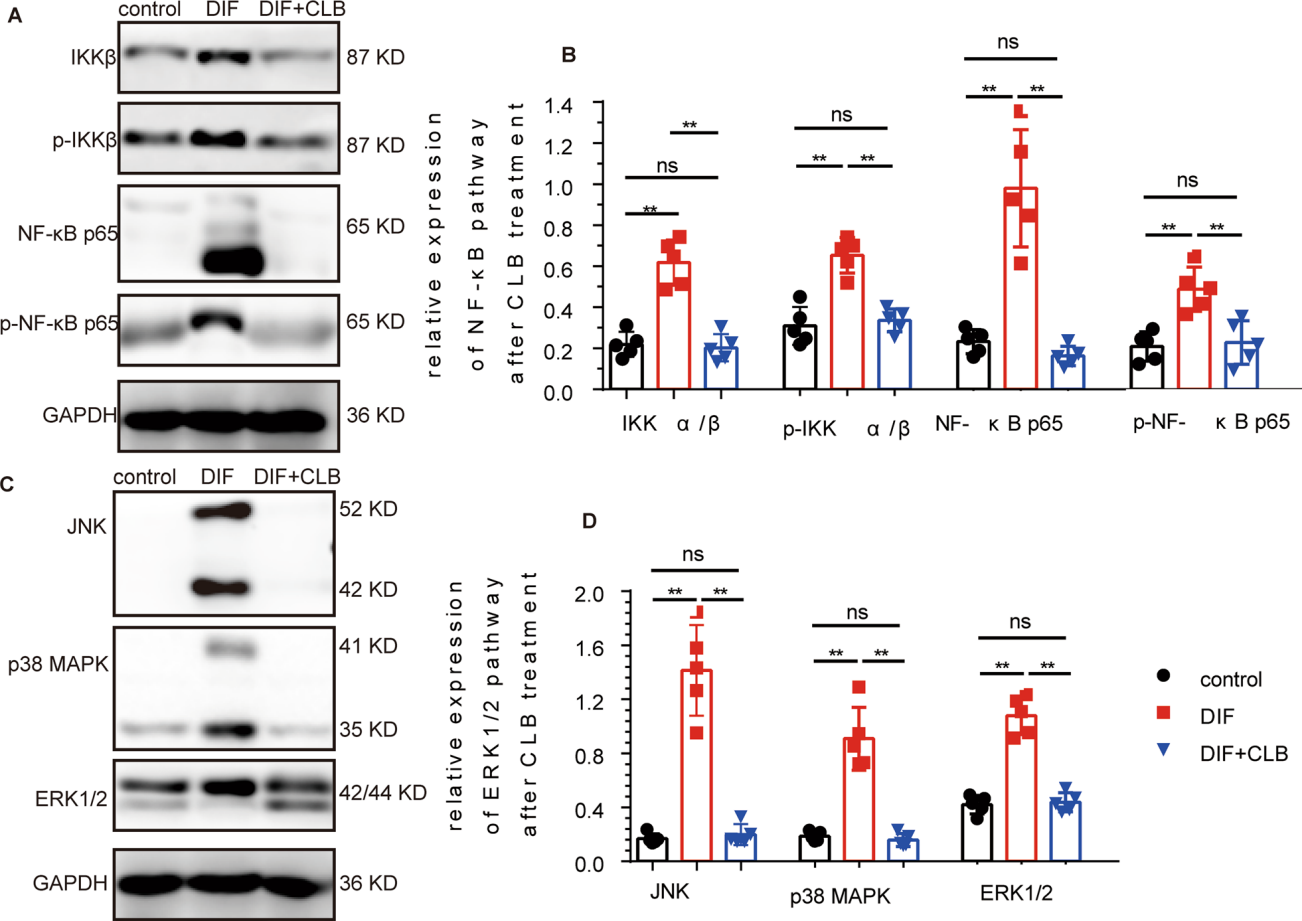
CLB-inhibited NF-κB and ERK1/2 signaling pathways. **A**, **B** IKKα/β, p-IKKα/β, NF-κB p65, and p-NF-κB p65 expressions were increased significantly after 48 h of differentiation and decreased after CLB (100 μM) treatment (*F* = 45.315, 9.154, 123.457, and 13.449, all *P* < 0.001). **C**, **D** The JNK, p38 MAPK, and ERK1/2 levels were increased significantly after 48 h of differentiation and decreased after CLB (100 μM) treatment (*F* = 247.631, 154.638, and 14.324, all *P* < 0.001)

After 48 h of myogenic differentiation, the expression levels of IKK α/β, p-IKK α/β, NF-κB p65, and NF-κB p-p65 of the NF-κB signaling pathway were significantly increased in C2C12 cells but were significantly inhibited by 100 μM CLB (Fig. 7A, B). Similarly, we assessed protein expressions in ERK1/2 signaling pathway and found that JNK, p38 MAPK, and ERK1/2 were significantly increased at 48 h after differentiation, while JNK, p38 MAPK, and ERK1/2 expressions were significantly inhibited at 48 h after 100 μM CLB treatment.

## Discussion

### The repair function is impaired by aging, and SCs undergo aging-related changes

While skeletal muscle easily regenerates, aging is inevitable, and the process of aging is mainly reflected by the decreased proportion of skeletal muscle cells and the gradual increase in the degree of fibrosis [8]. Upon injury, the repair of injured skeletal muscle is delayed and weakened over time [42]. Skeletal muscle cells are closely related to SCs, which activate, proliferate, and differentiate in response to injury and inflammatory factors and repair or fuse to injured muscle cells. However, aging may alter the SC microenvironment and niche [43]. The niche cannot maintain a balance, resulting in SC instability, loss of stemness, and ultimately a reduction in the number of SCs. We found that the number of SCs was decreased in the aging mice and that the number of Myf5(−) PAX7(+) quiescent SCs was further decreased.

### Sympathetic nerves play an important role in SCs and in skeletal muscle repair

In recent years, increasing attention has been paid to the effect of sympathetic nerves on stem cells. The role of sympathetic nerves in regulating mesenchymal stem cells and hematopoietic stem cells has been extensively explored [19, 44]. Sympathetic abnormalities participate in aging-related disorders [12], and sympathetic nerves may be considered for the future development of corresponding therapeutic strategies to regulate stem cells due to their adaptability [45]. Local sympathetic hyperinnervation of myofascial trigger point hinders the repair of skeletal muscle [23, 46]. The repair of myofascial trigger points was found to be ideal, and the number of SCs was increased after two injections of 6-ODHA [25]. However, complete CS cannot promote the repair of skeletal myofascial trigger points after modeling [24]. The sympathetic innervation of skeletal muscle in aging mice was decreased, and the repair of skeletal muscle was impaired after injury. Therefore, sympathetic nerves may regulate the stemness of SCs. We speculated that sympathetic nerves may be a component of the SC niche. Sympathetic hyperinnervation or hypoinnervation decreases the stability of SCs, and normal sympathetic nerve activity helps to maintain the stemness of SCs, which is conducive to skeletal muscle repair after injury. This provides a scientific basis for clinical intervention with SCs and a favorable basis for the clinical diagnosis and treatment of disorders such as age-related myopenia and myofascial pain syndrome. Clearly elucidating the underlying mechanisms will pave the way for targeting the sympathetic nerves for the treatment of aging [13].

### CLB, a β2-ADR agonist, partially rescues the sympathetic hypoinnervation induced by CS and aging-induced changes in muscle

β-ADR, mainly the β2 subtype, is expressed in skeletal muscle cells [40, 41]. Additionally, SCs were shown to express β-ADR [38], and we found β2 to be the main subtype expressed, with low expression of the β1 subtype and almost no expression of the β3 subtype. Moreover, the expression of NE was significantly decreased after CS. CLB promoted the expression of NE but did not significantly increase its expression in control mice. After injury, the NE expression in the CS-treated mice was increased significantly but not to the extent of that in the CS + CLB group after injury. After CS, the sympathetic innervation in young mice was substantially decreased and CLB partially rescued the sympathetic hypoinnervation and protected the normal activity of the sympathetic nerves. CLB enhanced the repair of skeletal muscle injury in the CS group and promoted the maintenance of the numbers of total, quiescent, and self-renewing SCs. These findings can potentially be attributed to the role of sympathetic nerves in maintaining the stemness of SCs through β2-ADR.

### CLB does not promote the proliferation of SCs in vitro but inhibits their myogenic differentiation, potentially via the NF-κB and ERK1/2 signaling pathways

CLB was found to maintain the numbers of SCs and quiescent SCs in vivo, while it did not promote the proliferation of SCs in vitro but inhibited their myogenic differentiation. These results suggest that CLB does not directly promote the proliferation of SCs but rather affects their protection, possibly by regulating the activity of sympathetic nerves and maintaining the stability of the SC microenvironment. Increased p38αβ MAPK activity was observed in aged SCs [47, 48], and CLB was shown to suppress the phosphorylation of ERK1/2 [49]. In our study, the expression of the main proteins in NF-κB and ERK1/2 signaling pathway was significantly increased in C2C12 cells after myogenic differentiation, which were significantly inhibited by CLB. Thus, CLB inhibits myogenic differentiation partly via the NF-κB and ERK1/2 signaling pathways.

## Conclusions

In conclusion, our study revealed potential mechanisms by which sympathetic nerves form the microenvironment or niche of SCs, which regulate the myogenic repair of SCs after injury and mediate the effects of SCs through β2-ADR. These findings could contribute to the development of novel strategies for promoting skeletal muscle injury repair and combating aging.

## Supplementary Information

**Additional file 1. Figure S1**. The ability to repair muscle injury is weakened in aged mice on days 10 and 21. (A, B) The difference in the number of myonuclei per muscle fiber (transverse section) was not significant among NC (young mice), CS, CLB, and CS+CLB groups (*F* = 1.99, *P* = 0.139). (C) The fibrotic area in the aged mice was larger than that in the young mice 10 days after injury (*n* = 8, *t* = 2.517, *P* = 0.025). (D) Representative figures show impaired repair of injured skeletal muscle in aged mice on days 10 and 21 after injury. (E) The fibrotic area in the aged mice was larger than that in the young mice 21 days after injury (*n* = 8, *t* = 2.041, *P* = 0.017). (F) The muscle cell area in the aged mice was smaller than that in the young mice 10 days after injury (*n* = 8, *t* = 2.623, *P* = 0.028). (G) The muscle cell area in the aged mice was smaller than that in the young mice 21 days after injury (*n* = 8, *t* = 3.019, *P* = 0.008). One-way ANOVA and Tukey’s test, ns, not significant, *P < 0.05, **P < 0.001.

**Additional file 2. Figure S2**. CS results in impaired repair of muscle injury, which can be reversed by CLB. (A) Representative SR and HE images of skeletal muscle injury in the injury group, CS+ injury group, and CS+ CLB+ injury group on days 3 and 21 after injury. (B) The number of myonuclei per muscle fiber (transverse section) was decreased significantly after CS (*P* = 0.003), which was rescued by CLB on day 21 after injury (*P* = 0.010). (C) Differences in the fibrotic area were nonsignificant among the injury, CS+ injury, and CS+ CLB+ injury on day 3 after injury (*n* = 8, *F* = 0.174, *P* = 0.513), and nonsignificant between the injury group and CS+ CLB+ injury group but significant among these three groups on day 21 (*n* = 8, *F* = 1.017, *P* = 0.207). (D) Differences in the muscle cell area were nonsignificant among the injury, CS+ injury, and CS+ CLB+ injury groups on day 3 after injury (*n* = 8, *F* = 0.459, *P* = 0.343), nonsignificant between the injury group and CS+ CLB+ injury group but significant among these three groups on day 21 (*n* = 8, *F* = 0.459, *P* = 0.343). One-way ANOVA and Tukey’s test, ns, not significant, *P < 0.05, **P < 0.001.

## Data Availability

All data generated or analyzed during this study are included in this published article.

## Abbreviations

6-OHDA: 6-Hydroxydopamine hydrochloride
ADR: Adrenoceptor
BaCl_2_: Barium chloride
CLB: Clenbuterol
CS: Sympathectomy
DMEM: Dulbecco’s modified Eagle’s medium
ELISA: Enzyme-linked immunosorbent assay
ERK1/2: Extracellular signal regulated kinase ½
FBS: Fetal bovine serum
JNK: Jun N-terminal kinase
MyoD: Myoblast determination protein
Myf5: Myogenic factor 5
MyoG: Myogenin
NE: Norepinephrine
NF-κB: Nuclear factor-κB
NPY: Neuropeptide Y
PAX7: Paired box protein 7
SC: Satellite cell
TA: Tibialis anterior
TH: Tyrosine hydroxylase
WB: Western blot

## References

[1] Mauro A (1961). Satellite cell of skeletal muscle fibers. J Biophys Biochem Cytol.

[2] Kuang S, Chargé SB, Seale P, Huh M, Rudnicki MA (2006). Distinct roles for Pax7 and Pax3 in adult regenerative myogenesis. J Cell Biol.

[3] Gayraud-Morel B, Chrétien F, Jory A, Sambasivan R, Negroni E, Flamant P (2012). Myf5 haploinsufficiency reveals distinct cell fate potentials for adult skeletal muscle stem cells. J Cell Sci.

[4] Lindström M, Pedrosa-Domellöf F, Thornell LE (2010). Satellite cell heterogeneity with respect to expression of MyoD, myogenin, Dlk1 and c-Met in human skeletal muscle: application to a cohort of power lifters and sedentary men. Histochem Cell Biol.

[5] Xin F, Hu L, Wenjun Y, Wanhong H, Yong Z, Ping H (2019). Regeneration of skeletal muscle. Chin J Cell Biol.

[6] Jin X, Kim JG, Oh MJ, Oh HY, Sohn YW, Pian X (2007). Opposite roles of MRF4 and MyoD in cell proliferation and myogenic differentiation. Biochem Biophys Res Commun.

[7] Kuang S, Kuroda K, Le Grand F, Rudnicki MA (2007). Asymmetric self-renewal and commitment of satellite stem cells in muscle. Cell.

[8] Lukjanenko L, Karaz S, Stuelsatz P, Gurriaran-Rodriguez U, Michaud J, Dammone G (2019). Aging disrupts muscle stem cell function by impairing matricellular WISP1 secretion from fibro-adipogenic progenitors. Cell Stem Cell.

[9] Etienne J, Liu C, Skinner CM, Conboy MJ, Conboy IM (2020). Skeletal muscle as an experimental model of choice to study tissue aging and rejuvenation. Skelet Muscle.

[10] Henze H, Jung MJ, Ahrens HE, Steiner S, von Maltzahn J (2020). Skeletal muscle aging—stem cells in the spotlight. Mech Ageing Dev..

[11] Baker SE, Limberg JK, Dillon GA, Curry TB, Joyner MJ, Nicholson WT (2018). Aging alters the relative contributions of the sympathetic and parasympathetic nervous system to blood pressure control in women. Hypertension.

[12] Dairman W (1972). Catecholamine concentrations and the activity of tyrosine hydroxylase after an increase in the concentration of tyrosine in rat tissues. Br J Pharmacol.

[13] Yamamoto Y, Morozumi T, Takahashi T, Saruta J, Sakaguchi W, To M, et al. Effect of high fat and fructo-oligosaccharide consumption on immunoglobulin A in saliva and salivary glands in rats. Nutrients. 2021;13:1252. 10.3390/nu13041252 PMC8070188 33920202

[14] Gonzalez-Montelongo M, Fountain SJ (2021). Neuropeptide Y facilitates P2X1 receptor-dependent vasoconstriction via Y1 receptor activation in small mesenteric arteries during sympathetic neurogenic responses. Vascul Pharmacol..

[15] Ramirez-Villafaña M, Saldaña-Cruz AM, Aceves-Aceves JA, Perez-Guerrero EE, Fajardo-Robledo NS, Rubio-Arellano ED, et al. Serum neuropeptide Y levels are associated with TNF-α levels and disease activity in rheumatoid arthritis. J Immunol Res. 2020;2020:8982163. 10.1155/2020/8982163 PMC7182972 32377539

[16] Ulum B, Mammadova A, Özyüncü Ö, Uçkan-Çetinkaya D, Yanık T, Aerts-Kaya F (2020). Neuropeptide Y is involved in the regulation of quiescence of hematopoietic stem cells. Neuropeptides.

[17] Maryanovich M, Zahalka AH, Pierce H, Pinho S, Nakahara F, Asada N (2018). Adrenergic nerve degeneration in bone marrow drives aging of the hematopoietic stem cell niche. Nat Med.

[18] Chartier SR, Mitchell S, Majuta LA, Mantyh PW (2018). The changing sensory and sympathetic innervation of the young. Adult Aging Mouse Femur Neurosci.

[19] Wang ZM, Rodrigues A, Messi ML, Delbono O (2020). Aging blunts sympathetic neuron regulation of motoneurons synaptic vesicle release mediated by β1- and α2B-adrenergic receptors in geriatric mice. J Gerontol A Biol Sci Med Sci.

[20] Voltarelli VA, Bechara LR, Bacurau AV, Mattos KC, Dourado PM, Bueno CJ (2014). Lack of β2-adrenoceptors aggravates heart failure-induced skeletal muscle myopathy in mice. J Cell Mol Med.

[21] Cao L, Gao Y, Wu K, Li Y, Chen C, Yuan S (2020). Sympathetic hyperinnervation in myofascial trigger points. Med Hypotheses.

[22] Wu K, Yuan S, Liao L, Li Y (2020). Effects of sympathetic nerve and muscle satellite cells on myofascial pain point repair. Chin J Pain Med.

[23] Yuan S, Liman Y, Kai W, Mingkui X, Yikai L, Yucong Z (2020). Effects of chemical sympathectomy on inflammation of myofascial trigger points and myogenic differentiation of muscle satellite cells. J Pract Med.

[24] Higashihara T, Nishi H, Takemura K, Watanabe H, Maruyama T, Inagi R (2021). β2-adrenergic receptor agonist counteracts skeletal muscle atrophy and oxidative stress in uremic mice. Sci Rep.

[25] Hauck JS, Howard ZM, Lowe J, Rastogi N, Pico MG, Swager SA, et al. Mineralocorticoid receptor signaling contributes to normal muscle repair after acute injury. Front Physiol. 2019;10:1324. 10.3389/fphys.2019.01324 PMC6830343 31736768

[26] Morton AB, Norton CE, Jacobsen NL, Fernando CA, Cornelison D, Segal SS (2019). Barium chloride injures myofibers through calcium-induced proteolysis with fragmentation of motor nerves and microvessels. Skelet Muscle.

[27] Mishra I, Pullum KB, Thayer DC, Plummer ER, Conkright BW, Morris AJ (2020). Chemical sympathectomy reduces peripheral inflammatory responses to acute and chronic sleep fragmentation. Am J Physiol Regul Integr Comp Physiol.

[28] Oben JA, Roskams T, Yang S, Lin H, Sinelli N, Li Z (2003). Sympathetic nervous system inhibition increases hepatic progenitors and reduces liver injury. Hepatology.

[29] Rudak PT, Gangireddy R, Choi J, Burhan AM, Summers KL, Jackson DN (2019). Stress-elicited glucocorticoid receptor signaling upregulates TIGIT in innate-like invariant T lymphocytes. Brain Behav Immun.

[30] Kim J, Grotegut CA, Wisler JW, Mao L, Rosenberg PB, Rockman HA (2020). The beta-arrestin-biased beta-adrenergic receptor blocker carvedilol enhances skeletal muscle contractility. Proc Natl Acad Sci USA.

[31] Spurlock DM, McDaneld TG, McIntyre LM (2006). Changes in skeletal muscle gene expression following clenbuterol administration. BMC Genomics.

[32] Motohashi N, Asakura Y, Asakura A. Isolation, culture, and transplantation of muscle satellite cells. J Vis Exp. 2014;(86):50846. 10.3791/50846 PMC4131689 24747722

[33] Hüttner SS, Ahrens HE, Schmidt M, Henze H, Jung MJ, Schüler SC (2019). Isolation and culture of individual myofibers and their adjacent muscle stem cells from aged and adult skeletal muscle. Methods Mol Biol.

[34] Cottle BJ, Lewis FC, Shone V, Ellison-Hughes GM (2017). Skeletal muscle-derived interstitial progenitor cells (PICs) display stem cell properties, being clonogenic, self-renewing, and multi-potent in vitro and in vivo. Stem Cell Res Ther.

[35] Chapman MR, Balakrishnan KR, Li J, Conboy MJ, Huang H, Mohanty SK (2013). Sorting single satellite cells from individual myofibers reveals heterogeneity in cell-surface markers and myogenic capacity. Integr Biol Quant Biosci Nano Macro.

[36] Chen S, Yue J, Zhang J, Jiang M, Hu T, Leng W, et al. Continuous exposure of isoprenaline inhibits myoblast differentiation and fusion through PKA/ERK1/2-FOXO1 signaling pathway. Stem Cell Res Ther. 2019;10(1):70. 10.1186/s13287-019-1160-x PMC6394105 30819239

[37] Motohashi N, Asakura A. Muscle satellite cell heterogeneity and self-renewal. Front Cell Dev Biol. 2014;2:1. 10.3389/fcell.2014.00001 PMC4206996 25364710

[38] Williams RS, Caron MG, Daniel K (1984). Skeletal muscle beta-adrenergic receptors: variations due to fiber type and training. Am J Physiol.

[39] Kim YS, Sainz RD, Molenaar P, Summers RJ (1991). Characterization of beta 1- and beta 2-adrenoceptors in rat skeletal muscles. Biochem Pharmacol.

[40] Oh J, Sinha I, Tan KY, Rosner B, Dreyfuss JM, Gjata O (2016). Age-associated NF-kappaB signaling in myofibers alters the satellite cell niche and re-strains muscle stem cell function. Aging (Albany NY).

[41] Garg K, Boppart MD (1985). Influence of exercise and aging on extracellular matrix composition in the skeletal muscle stem cell niche. J Appl Physiol.

[42] Aguila HL (2006). Regulation of hematopoietic niches by sympathetic innervation. BioEssays.

[43] Yu AQ, Wang J, Zhou XJ, Chen KY, Cao Y, Wang ZX (2020). Senescent cell-secreted netrin-1 modulates aging-related disorders by recruiting sympathetic fibers. Front Aging Neurosci.

[44] Price T, Sipkins DA (2014). Rewiring the niche: sympathetic neuropathy drives malignant niche transformation. Cell Stem Cell.

[45] Ge HY, Fernandez-de-las-Penas C, Arendt-Nielsen L (2006). Sympathetic facilitation of hyperalgesia evoked from myofascial tender and trigger points in patients with unilateral shoulder pain. Clin Neurophysiol.

[46] Balasubramanian P, Hall D, Subramanian M (2019). Sympathetic nervous system as a target for aging and obesity-related cardiovascular diseases. Geroscience.

[47] Bernet JD, Doles JD, Hall JK, Kelly TK, Carter TA, Olwin BB (2014). p38 MAPK signaling underlies a cell-autonomous loss of stem cell self-renewal in skeletal muscle of aged mice. Nat Med.

[48] Troy A, Cadwallader AB, Fedorov Y, Tyner K, Tanaka KK, Olwin BB (2012). Coordination of satellite cell activation and self-renewal by Par-complex-dependent asymmetric activation of p38α/β MAPK. Cell Stem Cell.

[49] Chen M, Liu C, Wang M, Wang H, Zhang K, Zheng Y (2017). Clenbuterol induces cell cycle arrest in C2C12 myoblasts by delaying p27 degradation through β-arrestin 2 signaling. Int J Biol Sci.

