# Supplementary figures and images for: Sympathetic activity is correlated with satellite cell aging and myogenesis via β2-adrenoceptor
Source: Stem Cell Res Ther. 2021 Sep 16;12:505. doi: 10.1186/s13287-021-02571-8 (PMC8447727; doi:10.1186/s13287-021-02571-8)

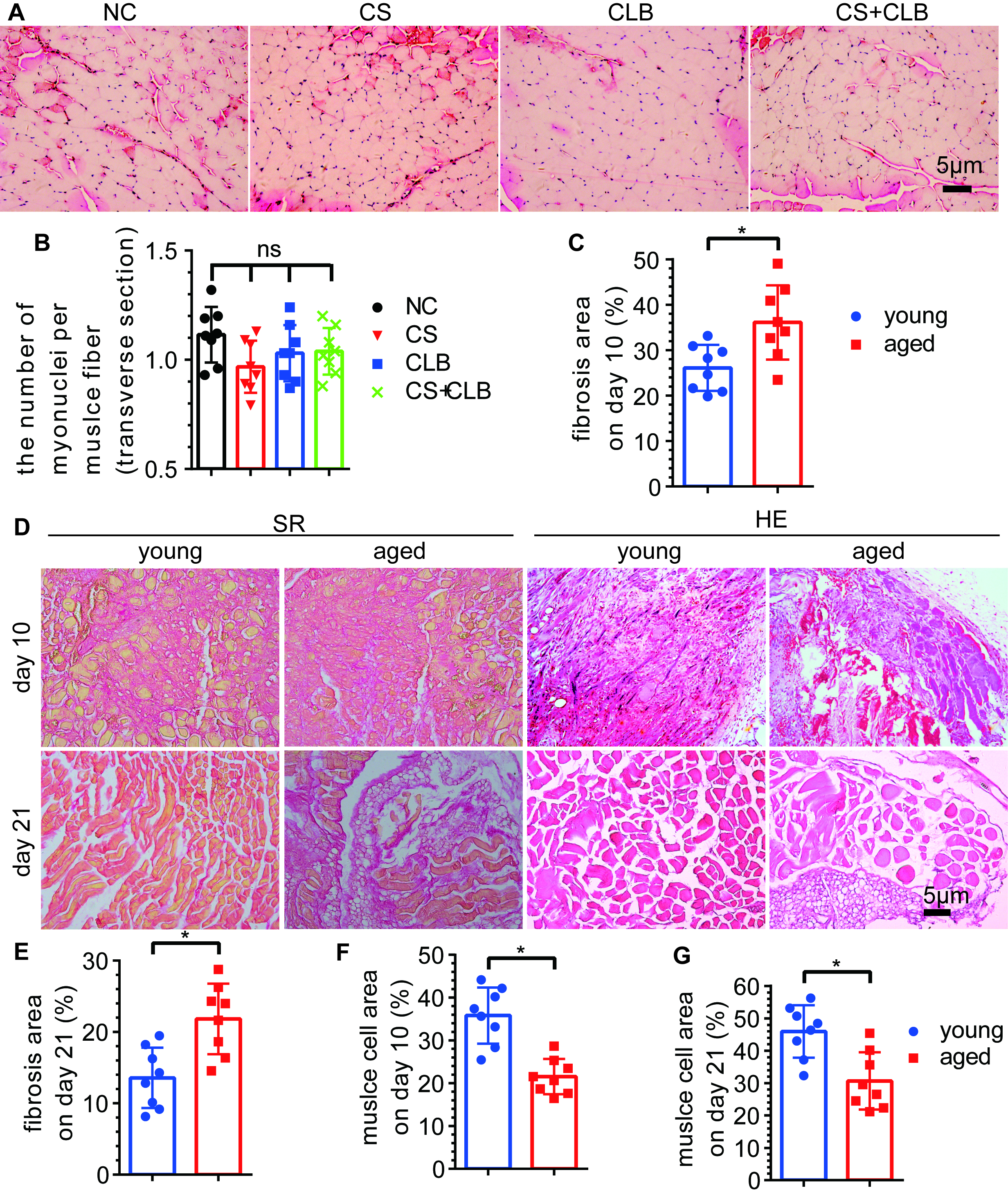

Supplement: Supplementary file 1 — Additional file 1. Figure S1. The ability to repair muscle injury is weakened in aged mice on days 10 and 21. (A, B) The difference in the number of myonuclei per muscle fiber (transverse section) was not significant among NC (young mice), CS, CLB, and CS+CLB groups (F = 1.99, P = 0.139). (C) The fibrotic area in the aged mice was larger than that in the young mice 10 days after injury (n = 8, t = 2.517, P = 0.025). (D) Representative figures show impaired repair of injured skeletal muscle in aged mice on days 10 and 21 after injury. (E) The fibrotic area in the aged mice was larger than that in the young mice 21 days after injury (n = 8, t = 2.041, P = 0.017). (F) The muscle cell area in the aged mice was smaller than that in the young mice 10 days after injury (n = 8, t = 2.623, P = 0.028). (G) The muscle cell area in the aged mice was smaller than that in the young mice 21 days after injury (n = 8, t = 3.019, P = 0.008). One-way ANOVA and Tukey’s test, ns, not significant, *P < 0.05, **P < 0.001. [file 13287_2021_2571_MOESM1_ESM.tif]

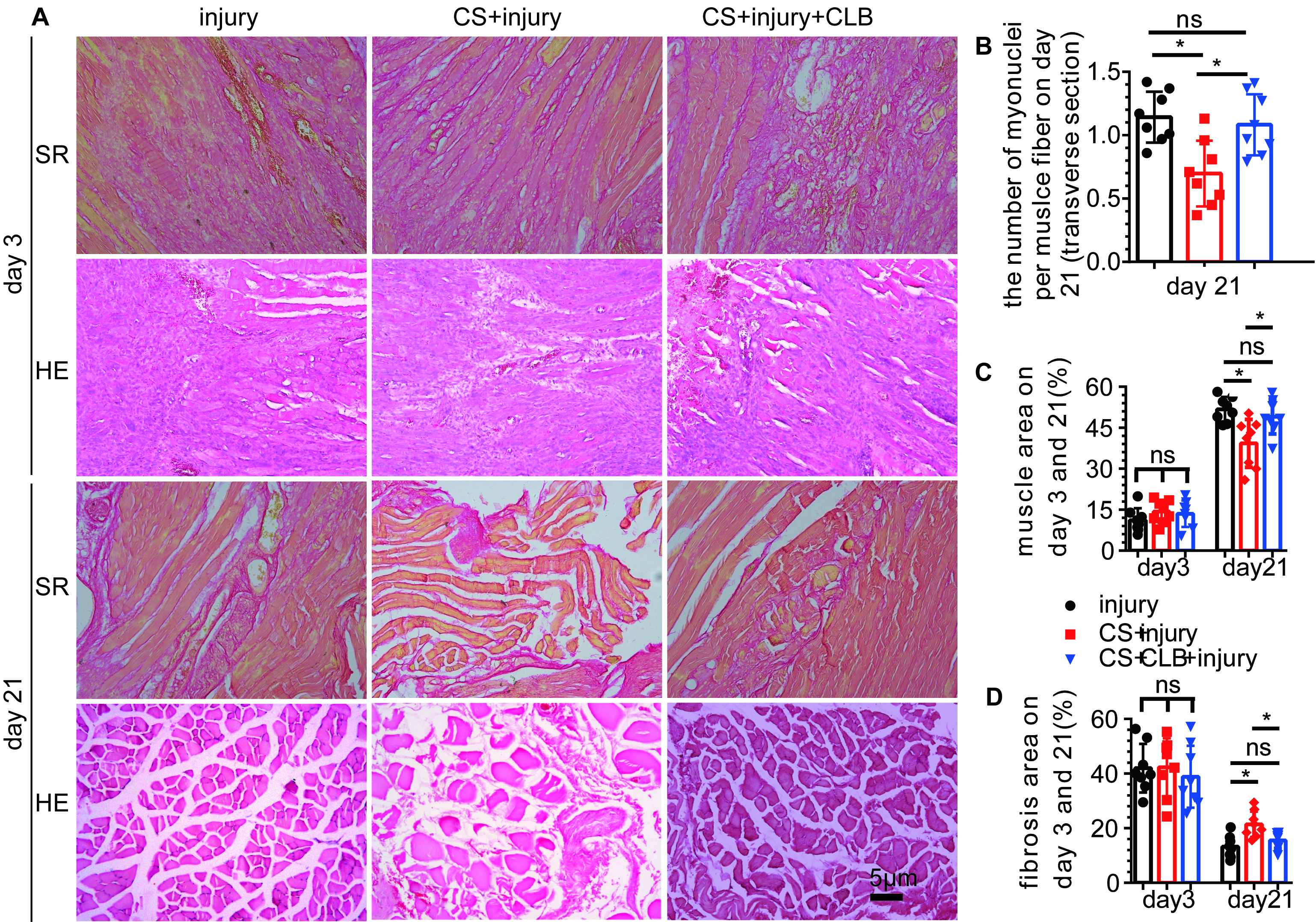

Supplement: Supplementary file 2 — Additional file 2. Figure S2. CS results in impaired repair of muscle injury, which can be reversed by CLB. (A) Representative SR and HE images of skeletal muscle injury in the injury group, CS+ injury group, and CS+ CLB+ injury group on days 3 and 21 after injury. (B) The number of myonuclei per muscle fiber (transverse section) was decreased significantly after CS (P = 0.003), which was rescued by CLB on day 21 after injury (P = 0.010). (C) Differences in the fibrotic area were nonsignificant among the injury, CS+ injury, and CS+ CLB+ injury on day 3 after injury (n = 8, F = 0.174, P = 0.513), and nonsignificant between the injury group and CS+ CLB+ injury group but significant among these three groups on day 21 (n = 8, F = 1.017, P = 0.207). (D) Differences in the muscle cell area were nonsignificant among the injury, CS+ injury, and CS+ CLB+ injury groups on day 3 after injury (n = 8, F = 0.459, P = 0.343), nonsignificant between the injury group and CS+ CLB+ injury group but significant among these three groups on day 21 (n = 8, F = 0.459, P = 0.343). One-way ANOVA and Tukey’s test, ns, not significant, *P < 0.05, **P < 0.001. [file 13287_2021_2571_MOESM2_ESM.tif]
